# Rapid detection of* Mycoplasma mycoides* subsp. *capri* and *Mycoplasma capricolum* subsp. *capripneumoniae* using high-resolution melting curve analysis

**DOI:** 10.1038/s41598-021-93981-4

**Published:** 2021-07-28

**Authors:** Jing-peng Zhang, Zhi-cheng Liu, Jin-xiu Jiang, Yu-sheng Lin, Wei You, Qi-lin Hu

**Affiliations:** 1 Institute of Animal Husbandry & Veterinary Medicine, Fujian Academy of Agricultural Sciences, Fuzhou, 350013 China; 2 Institute of Animal Health, Guangdong Academy of Agricultural Sciences, Guangzhou, 510640 China

**Keywords:** Microbiology, Infectious-disease diagnostics

## Abstract

*Mycoplasma capricolum* *subsp.*subsp. *capripneumonia* (Mccp) and *Mycoplasma mycoides* *subsp.*sbusp. *capri* (Mmc) cause caprine pleuropneumonia (CCPP) and mycoplasmal pneumonia in goats and sheep (MPGS), respectively. These diseases cannot be identified on clinical symptoms alone and it is laborious to distinguish them using biochemical methods. It is therefore important to establish a simple, rapid identification method for Mccp and Mmc. Here, we report a high-resolution melting (HRM) curve analysis using specific primers based on the Mmc 95010 strain *MLC_0560* and Mccp F38 strain *MCCPF38_00984* gene sequences. The method was highly specific with intra- and inter-batch coefficients of variation < 1%. The lower limit of detection for Mccp and Mmc was 55 copies/μL and 58 copies/μL, respectively. HRM and fluorescence qPCR results were compared using 106 nasal swabs and 47 lung tissue samples from goats (HRM-qPCR coincidence rate 94.8%; 145/153). Mycoplasma isolation and identification was performed on 30 lung tissue samples and 16 nasal swabs (HRM-culturing coincidence rate 87.0%; 40/46). HRM analysis was more sensitive than fluorescence qPCR and Mycoplasma isolation, indicating the practicality of HRM for accurate and rapid identification of Mccp and Mmc, and diagnosis and epidemiology of CCPP and MPGS.

## Introduction

*Mycoplasma capricolumsubsp *subsp. *capripneumonia* (Mccp) is the causative pathogen of contagious caprine pleuropneumonia (CCPP). Clinically, the disease is characterized by hyperpyrexia, rhinorrhea, cough, cellulose pleuropneumonia, abortion, and progressive emaciation of some ewes^1^. The incidence can reach 80–100%, and the mortality rate can reach 60–80%^2,3^. CCPP is one of the statutory reportable animal infectious diseases listed by the World Organization for Animal Health^4^. *Mycoplasma mycoides* subsp. *capri* (Mmc) is one of the causative pathogens of mycoplasmal pneumonia of goats and sheep (MPGS)^5^. The incidence of MPGS caused by Mmc can reach 50%, and the mortality rate can reach 40%^6,7^. In addition to causing pathological lung changes similar to Mccp in goats, Mmc can also cause mastitis, arthritis, and keratitis, among other conditions^8^, causing significant economic losses to the goat farming industry. Clinical symptoms and necropsy lesions of CCPP and MPGS are similar, and distinguishing them clinically is difficult. Mccp and Mmc are both members of the *M. mycoides* cluster and show high genetic and antigen similarity^9^. In fact, in China they have been traditionally referred to as CCPP collectively. However, only Mccp has been shown to conform to Koch’s postulate, and it is the only causative pathogen of CCPP^4^. Laboratory identification is typically conducted by mycoplasma pathogen isolation, culture, and identification combined with serological testing, but this method can only be carried out in a laboratory, is difficult to standardize, and is time-consuming, labor-intensive, and lacks proper biological safeguards. Therefore, it is important to establish a rapid identification method for Mccp and Mmc.

Established molecular detection methods for Mccp and Mmc mainly include PCR detection^10–12^, multiplex PCR^13–15^, nested PCR for Mmc specifically^16^, and real-time fluorescence quantitative PCR (qPCR) detection^17–19^. A high-resolution melting (HRM) curve is useful for detecting genetic mutations. The technique is based on the use of a novel saturated fluorescent dye first proposed by the Wittwer Laboratory of the University of Utah^20^. This method involves genotyping by monitoring the binding of double-stranded DNA with the fluorescent dye and PCR amplification products in real-time during the heating process according to the differences in fragment length, G + C content, and nucleic acid distribution^21^. It shows strong specificity and high sensitivity and is suitable for establishing Mccp and Mmc identification methods.

No HRM-based methods have been developed to identify Mccp and Mmc. Therefore, in this study, bioinformatics software was used to analyze the genome sequences of Mccp and Mmc to screen for conserved fragments with adequate differences to design primers that can distinguish the two microbes. The HRM detection method described here was established using the small-fragment amplification method in order to rapidly detect and identify Mccp and Mmc.

## Materials and methods

### Mycoplasmas, virus strains, bacterial strains, and clinical samples

Referring to Lin et al.^22^, the strains used in this study were: Mccp strain F38, Mccp strain California Kid, Mmc strain PG3, were provided by Prof. Yuefeng Chu at Lanzhou Veterinary Research Institute, Chinese Academy of Agricultural Sciences (LVRI, CAAS). The *M*. *leachii* PG50 strain was a gift from Prof. Jiuqing Xin at Harbin Veterinary Research Institute, CAAS (HVRI, CAAS). Mmc strain Y-goat and *M. agalactiae* strain PG2 were gifts from Prof. Cheng Tang at the College of Life and Technology, Southwest University for Nationalities. *E. coli* strain ATCC25922 (Guangdong Huankai Microbial), Pm strain CVCC44801 (China Institute of Veterinary Drug Control), and Sa strain 261111 (National Institutes for Food and Drug Control) were gifts from Prof. Long-Fei Cheng, Institute of Animal Science and Veterinary Medicine, Fujian Academy of Agricultural Sciences. *Mannheimia haemolytica*, *Mycoplasma arginini*, *Acholeplasma laidlawii*, *M. bovis*, *Mycoplasma ovipneumoniae* (Movi), Mmc strain FJ-GT, Mmc strain FJ-CL, and *Orf virus* (ORFV) were all isolated, identified, and preserved in our laboratory. 153 clinical samples (106 nasal swabs and 47 lung tissue samples) of goats with clinically suspected MPGS were collected from the cities of Fuzhou, Ningde, Quanzhou, Putian, Sanming, and Nanping of Fujian Province between September 2014, and December 2018. All sample manipulations were performed in BSL-2 laboratories.

### Reagents

MiniBEST Bacteria Genomic DNA Extraction Kit Ver. 3.0, MiniBEST Viral RNA/DNA Extraction Kit Ver. 5.0, MiniBEST Plasmid Purification Kit Ver. 4.0, EXTAQ, MiniBEST Agarose Gel DNA Extraction Kit Ver. 4.0, and pMD ^TM^ 18-T Vector Cloning Kit were purchased from TAKARA Biomedical Technology (Beijing, China). Syto9 was purchased from ThermoFisher Scientific (Waltham, MA, USA).

### Primer design

**Figure 1:**
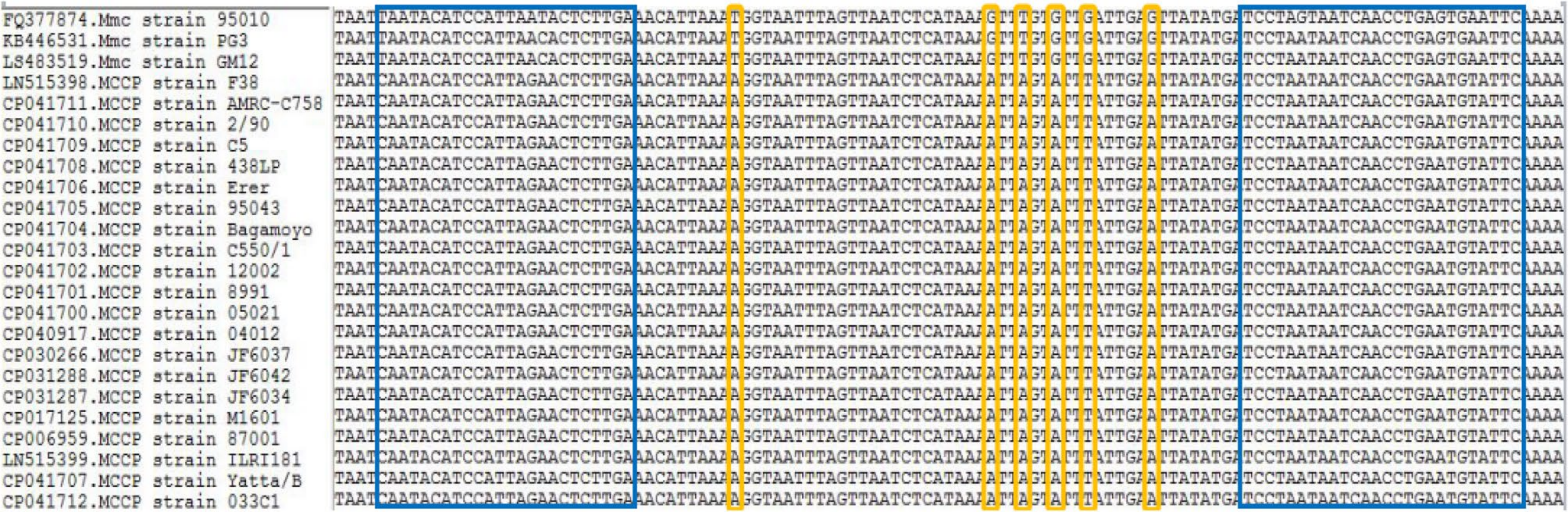
The design of primers for HRM. Conservation of the corresponding sequence of each primer was analysed using BioEdit sequence alignment editor v7.1.7 aligned, The corresponding sequence of each primer in all selected Mmc and Mccp isolates shown in bule colour, The different bases in all selected Mmc and Mccp isolates shown in yellow colour.

The sequences of the conserved gene *MLC_0560* of Mmc 95010 strain (FQ377874.1) and *MCCP* F38_*00984* (LN515398.1) from Mccp F38 were retrieved from GenBank. Using BioEdit Sequence Alignment Editor software for multiple sequences alignment (Fig. 1), a pair of specific primers was designed using Primer Premier 6.0 software. The primer sequences selected were: MF-107: 5′-CAATACATCCATTAGAACTCTTGA-3′; and MR-107: 5′-GAATACATTCAGGTTGATTATTAGGA-3′. Primers were synthesized by Sangon Biotech Co., Ltd. (Shanghai, China) targeted sequence length 107 bp. Melting temperature (Tm) of the sequence was calculated using uMELT online resource (https://www.dna.utah.edu/umelt/umelt.html).

### DNA extraction

All genomic DNAs were extracted using the MiniBEST Bacteria Genomic DNA Extraction Kit Ver. 3.0 according to manufacturer’s instructions, Viral DNAs was extracted with the MiniBEST Viral RNA/DNA Extraction Kit Ver. 5.0 according to manufacturer’s instructions. After DNA extraction, the concentration of each sample was measured with a UV–Vis spectrophotometer NanoDrop 2000 (Thermo Fisher, Wilmington, USA).

### Construction of a positive plasmid standard

Mmc and Mccp genomic DNA were used as the templates and the primers MF-107 and MR-107 were used for PCR amplification of the target fragment from those templates. The amplified products were recovered and purified after agar gel electrophoresis, and the purified target gene was ligated into the pMD18-T cloning vector. DH5α competent cells were transformed with the vector, after which the MiniBEST Plasmid Purification Kit Ver. 4.0 was used to extract the plasmid for PCR identification and restriction enzyme digestion identification. Agarose gel electrophoresis revealed a band of approximately 107 bp, consistent with the expected fragment size. Sequencing results from transformed bacteria were compared and analyzed using BLAST. The results showed that the homology of the Mmc PCR amplification products with Mmc PG3 strains was 100% and the homology of Mccp PCR amplification products with Mccp F38 strains was also 100%. This plasmid was then used as the positive control for subsequent experiments. The concentration was determined with a NanoDrop 2000 and converted into a copy number (number of copies = (amount × 6.022 × 10^23^)/(length × 1 × 10^9^  × 650)). The plasmids were stored at − 70 ℃ until use.

### Preliminary establishment of an HRM detection method

The reaction mix consisted of the following: the primers MF-107 and MR-107 (0.2 μmol/L), 2 μL template DNA, 12.5 μL premix Taq, 1.25 µmol/L Syto9, and ddH_2_O to a final volume of 25 μL. The thermocycler sequence used was as follows: pre-denaturation at 95 °C for 5 min followed by 40 cycles of denaturation at 95 °C for 30 s, annealing at 60 °C for 30 s, and extension at 72 °C for 20 s. HRM analysis was performed using a Rotor-Gene Q Fluorescence qPCR instrument. The detection conditions were as follows: denaturation at 95 °C for 5 min, renaturation at 45 °C for 1 min, and thermal equilibration at 50 °C for 10 s. From 50 °C, the melting curve was collected at a heating rate of 0.3 °C/s to 95 °C.

### Optimization of the HRM detection method

Annealing temperature optimization was performed by running scans at the following annealing temperatures based on the estimated Tm: 50 °C, 52 °C, 54 °C, 55 °C, 57 °C, 59 °C. 53 °C was chosen for subsequent experiments. After annealing temperature optimization, we performed primer concentration optimization. Reactions were performed with primer concentrations of 0.2 µmol/L, 0.4 µmol/L, 0.6 µmol/L, and 0.8 µmol/L. Based on these results, the optimum concentration was used for subsequent experiments.

### Specificity

Mmc, Mccp, Movi, ORFV, *E. coli*, *Salmonella*, *Pasteurella*, and *M. arginine* genomic DNA were extracted using the MiniBEST Bacteria Genomic DNA Extraction Kit Ver. 3.0 and used as templates to determine the specificity of the HRM method, the protocol according to “Optimization of the HRM detection method” section. If no specific amplification curve was generated and no expected Tm values, the samples were considered as negative cases.

### Sensitivity

The positive plasmid standard and Mmc and Mccp genomic DNA were diluted in tenfold serial dilutions with ddH_2_O, and PCR-HRM analysis was conducted under the optimal reaction conditions described in “Optimization of the HRM detection method” section to establish the minimum detection limit of the HRM method. Each dilution series, along with a blank control, was repeated three times. A standard curve diagram was drawn according to the Ct values and concentration. Ct values and melting curves were used to define the limit of detection (LOD). Data analysis and standard curve was performed with Rotor-Gene Q Series Software. For comparison with the conventional PCR assay, primers and reaction conditions used were the same as for the PCR step of the HRM method.

### Reproducibility

The positive control plasmid was diluted to three gradients to determine the reproducibility of the HRM method. All measurements were repeated three times for each sample at each concentration in the batch; the inter-batch test was carried out three times at an interval of 3 days. The HRM reaction system and cycle parameters are as described in “Optimization of the HRM detection method” section. The intra- and inter-batch coefficients of variation were calculated according to the Tm values of the two pathogens. Statistical analysis was performed with SPSS Statistics 25. Tm values between the two groups were compared with the independent t-test. We considered two-sided P values of 0.05 to be significant.

### DNA sequencing

To benchmark *Mmc* and *Mccp* detection with HRM analysis, DNA sequencing was performed. After the HRM reaction was completed, the HRM products were subjected to agarose gel electrophoresis, recovered, and purified. The purified target fragment was ligated into pMD 18-T and used to transform DH5α competent cells as described in “Construction of a positive plasmid standard” section. MF-107 and MR-107 were used for PCR to determine the positive clones, which sequenced at Takara Biotechnology (Dalian, China).

### Mycoplasma isolation and identification

Lung tissues were homogenized with modified Hayfick's culture medium. Ten-fold serial dilutions were prepared by combining 0.3 mL of the homogenized supernatant and subsequent dilutions with 2.7 mL of modified Hayfick's culture medium resulting in a dilution series from 10^–3^ to 10^–1^. All dilutions were incubated at 37 °C in 5% CO_2_, and three blind passages were performed every 7 days. Afterwards, 0.1 mL of the cultures that turned yellow was diluted tenfold to 10^–5^ to 10^–3^, and 0.2 mL of each dilution were streaked onto FRIIS Agar plates and cultured at 37 °C in 5% CO_2_ for 5–10 days. A single characteristic colony was inoculated in liquid culture in Modified Hayflick’s Medium, Mccp and Mmc field strains were identified using PCR methods^22^.

### Clinical sample testing

DNA was extracted from 153 clinical samples using the MiniBEST Bacteria Genomic DNA Extraction Kit Ver. 3.0. Samples were analyzed using the optimized HRM method in parallel with fluorescence qPCR^19^. Concurrently, detection using *Mycoplasma* isolation and identification described in “Mycoplasma isolation and identification” section was performed on 46 clinic samples (30 lung tissue samples and 16 nasal swabs). To evaluate the accuracy of the HRM method, samples with different test results between the three methods were sequenced.

### Ethics statement

The research reported here was approved by the Animal Care and Use Committee of the Institute of Animal Husbandry and Veterinary Medicine, Fujian Academy of Agricultural Sciences. For all clinical samples, we obtained informed consent of the goat’s owner. All methods were performed in accordance with the relevant guidelines and regulations.

## Results

### Optimization of HRM reaction conditions

Optimized HRM assay conditions were optimized as follows. The optimized primer concentrations were 0.4 μmol/L and the overall reaction mix contained 1× premix Taq, 1.25 μmol/L Syto9, 1 μL template, and ddH_2_O to a final volume of 25 μL. The optimal annealing temperature was determined to be 53 °C; therefore, the reaction conditions were: pre-denaturation at 95 °C for 3 min followed by 40 cycles of 95 °C for 15 s, 53 °C for 15 s, and 72 °C for 10 s. The HRM analysis procedure was as follows: 95 °C for 1 min, 45 °C for 1 min, 50 °C for 10 s, and heating to 90 °C at 0.3 °C/s.

### HRM specificity

**Figure 2:**
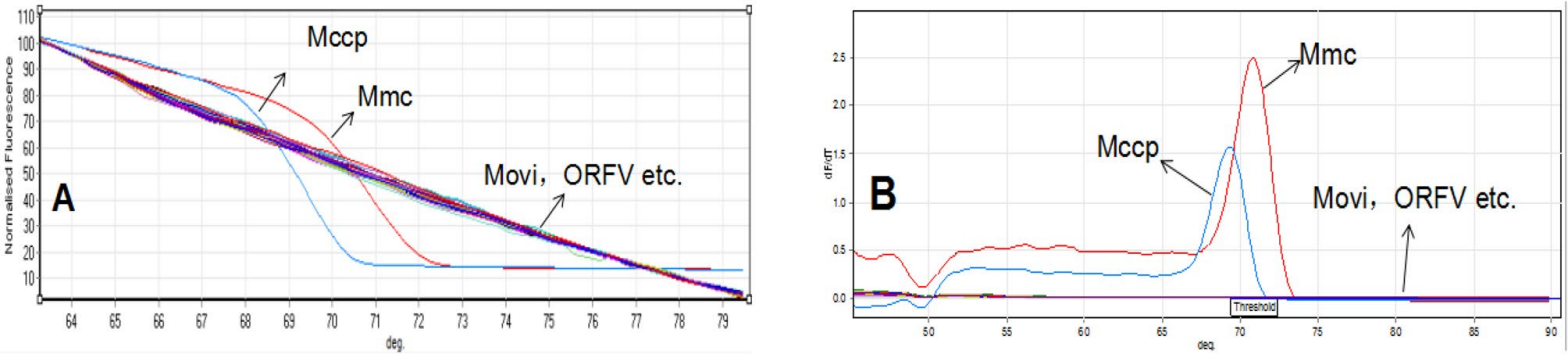
Specificity test of the HRM method for Mccp and Mmc. The pictures are generated by Rotor Gene Q. (**A**) Normalized characteristic curves of sample; (**B**) Melting curve of sample.

We next used the HRM method to detect Mmc PG3 strains and Mccp F38 strains. The results (Fig. 2) showed that only two specific melting peak curves of Mmc and Mccp were formed. The difference in Tm value of two dissolution curves was approximately 1.3, indicating that the two strains could easily be distinguished based on the melting curves, while no specific melting curves were generated from common goat pathogens as Movi and ORFV, indicating that this HRM method was specific for Mmc and Mccp.

### HRM reproducibility

As shown in Table 1, the intra- and inter-batch coefficients of variation for melting peak Tm1 were both within 0.08–0.11%, while for Tm2, they were 0.08–0.14% and 0.11–0.14%, respectively. As these values were all < 1%, the reproducibility of this method was verified. Tm values between the two groups were verified as statistically significant by the independent t-test (P = 0.000; P <0.01) (Table 1).

**Table 1:** Reproducibility assay of the HRM method.

No.	Intra-assay reproducibility				Inter-assay reproducibility			
	Tm1 mean $\overline{\upchi }$ ± SD	CV/%	Tm2 mean $\overline{\upchi }$ ± SD	CV/%	Tm1 mean $\overline{\upchi }$ ± SD	CV/%	Tm2 mean $\overline{\upchi }$ ± SD	CV/%
1 × 10^–2^	69.53 ± 0.06^B^	0.08	71.04 ± 0.09^A^	0.14	69.47 ± 0.06^B^	0.08	71.08 ± 0.08^A^	0.11
1 × 10^–3^	69.46 ± 0.06^B^	0.08	70.95 ± 0.09^A^	0.12	69.48 ± 0.08^B^	0.11	70.98 ± 0.08^A^	0.11
1 × 10^–4^	69.41 ± 0.08^B^	0.11	70.93 ± 0.06^A^	0.08	69.53 ± 0.06^B^	0.08	71.01 ± 0.1^A^	0.14

Different uppercase letters in the same row mean extremely significant difference (P = 0.000 < 0.01).

### HRM sensitivity

**Figure 3:**
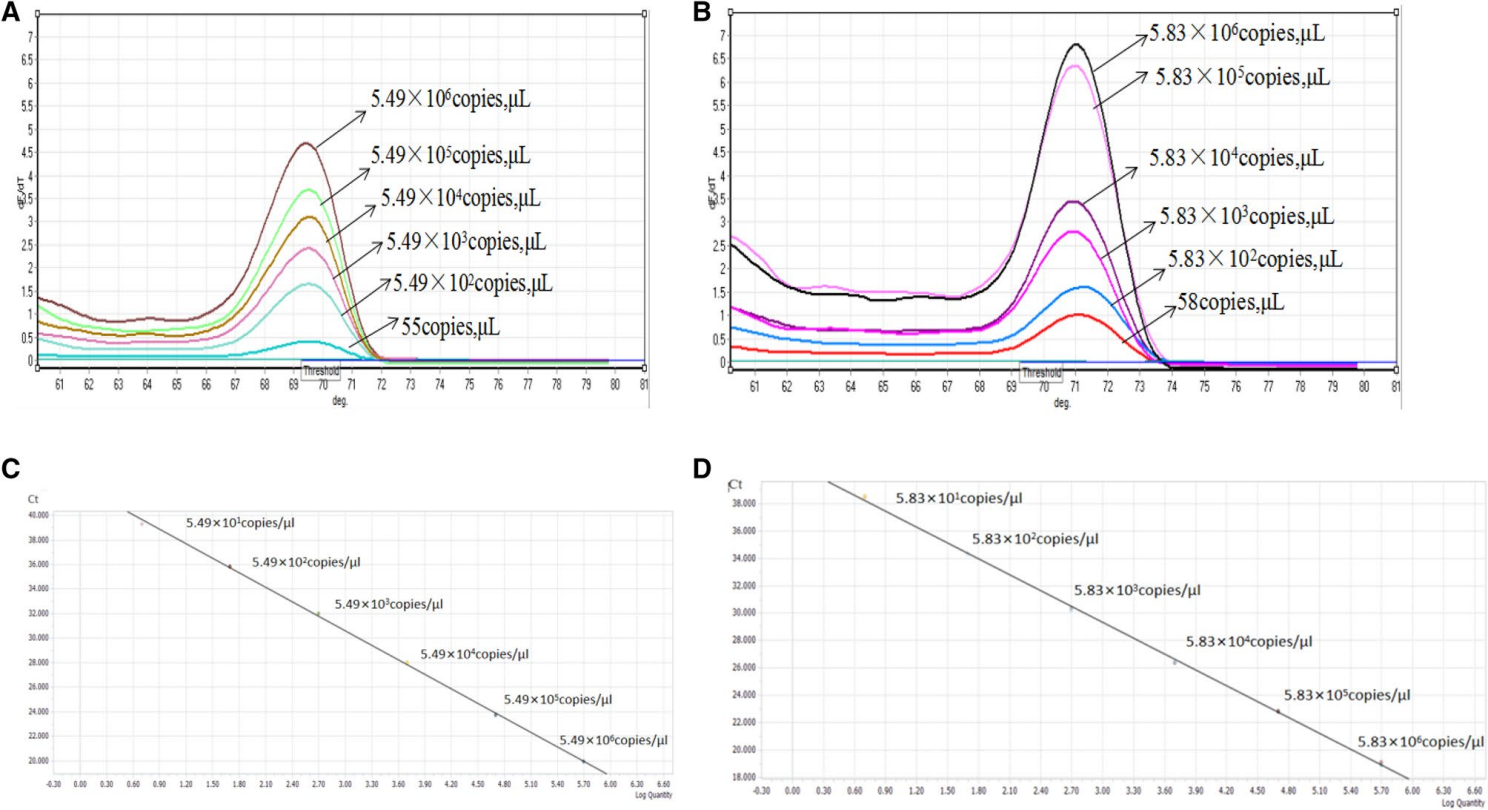
Sensitivity tests of the HRM method of *Mccp* and *Mmc* positive samples. The pictures are generated by Rotor Gene Q. (**A**) Sensitivity tests of the HRM method for *Mccp*; (**B**) Sensitivity tests of the HRM method for *Mmc*. (**C**) Standard curve of *Mccp* performed in a linear graph with R^2^ = 0.9972. (**D**) Standard curve of *Mmc* performed in a linear graph with R^2^ = 0.9974.

The positive control plasmids were diluted tenfold and detected using the HRM method described here as well as conventional PCR. The results (Fig. 3) indicated that specific melting curves and Tm values could be determined at Mccp and Mmc plasmid concentrations as low as 55 copies/μL and 58 copies/μL, respectively. The Ct values of Mccp and Mmc were both less than 40, there was a good correlation between copy number (10^5^ to 10^1^ copies/μL) and the Ct values, no reaction was detected at concentrations of 10^0^copies/μL, Taken together, the minimum detectable limits of the HRM method for Mccp and Mmc were 55 and 58 copies/μL, respectively. The formula between Ct values and copy number (10^5^ to 10^1^ copies/μL) for Mccp and Mmc is y = − 3.5765logx + 44.83 (R2 = 0.9972) and y = − 3.6640logx + 44.41 (R2 = 0.9974) respectively(Fig. 3). The minimum detectable Ct values between the two groups were compared with independent t-test, the statistical analyses show the average Ct differences between the two pathogens were significant (P = 0.012, P  < 0.05) (Table 2). The results of agarose gel electrophoresis showed that the corresponding minimum detection limits of conventional PCR were 5.49 × 10^4^ copies/μL for Mccp and 5.83 × 10^4^ copies/μL for Mmc, indicating that the HRM method was more sensitive than conventional PCR detection. This analysis was conducted using both the control plasmids and genomic DNA as the template. After the genomic DNA was diluted 10 times, the minimum detectable limit for Mmc and Mccp was determined as 300 fg and 400 fg, respectively.

**Table 2:** Comparison of LOD in the detection of Mmc and Mccp.

No.	Mccp		Mmc	
	Ct mean $\overline{\upchi }$ ± SD	CV/%	Ct mean $\overline{\upchi }$ ± SD	CV/%
10^–9^	38.81 ± 0.03^a^	0.09	38.36 ± 0.18^b^	0.4

Different lowercase letters in the same row mean significant difference (0.01 < P < 0.05). The average Ct values differences between the two pathogens were significant (P = 0.012 < 0.05).

### Clinical sample testing results

A total of 153 clinical samples from goats suspected of having mycoplasma infection were detected using HRM, real-time PCR, and pathogen isolation to compare the accuracy of the HRM method. The results (Table 3) showed that 31 Mmc positive samples were detected by HRM, but no Mccp were detected. A total of 23 Mmc HRM-positive samples were also detected with fluorescence qPCR while qPCR did not detect any *Mccp* in the samples. Using pathogen isolation methods, two Mmc positive samples were identified, while Mccp was not detected. Two samples positive for Mmc using mycoplasma isolation and identification were also positive for Mmc using the HRM and qPCR methods. Of the 31 HRM-positive samples for Mmc, only 23 were also determined positive with qPCR. The 8 samples that tested positive for Mmc by HRM but negative for Mmc by qPCR were sequenced, and the results were subjected to a BLAST search, which showed that the homology with Mmc was 100%. Thus, the coincidence rate of HRM and real-time PCR was 94.8% (145/153) and for HRM and pathogen isolation was 87.0% (40/46) (Table 3). Thus, the HRM method established in this study was more sensitive than fluorescence qPCR and pathogen isolation.

**Table 3:** Results of clinical samples with real-time PCR and HRM methods.

Detecting method		HRM		Total
		*Mmc* positive number	*Mmc* negative number	
Real-time PCR	Mmc positive number	23	0	23
	Mmc negative number	8	122	130
Sum		31	122	153
Pathogen isolation and identification	Mmc positive number	2	0	2
	Mmc negative number	6	38	44
Sum		8	38	46

## Discussion

Contagious caprine pleuropneumonia and mycoplasmal pneumonia in sheep and goats have been common in China for a long period of time. However, before 2007, mycoplasmal pneumonia in sheep and goats caused by Mmc was also regarded as contagious caprine pleuropneumonia in China^23–25^. This was mainly because Mccp and Mmc both belong to the *M. mycoides* cluster and have high homology and cause similar clinical symptoms. They show cross-reactivity in serology analysis making distinguishing these mycoplasmas with immunological methods difficult. Therefore, it is clinically important to establish a rapid and simple method for differentiating Mccp and Mmc.

Amores et al. have demonstrated that PCR is a faster and more sensitive method for detection of Mmc than mycoplasma isolation and identification^26^. Fluorescence qPCR is more rapid with greater sensitivity and specificity than conventional PCR, and has been widely used in the clinical detection of various pathogens^27–29^. Woubit^30^ established in 2007 a real-time PCR method for detecting the *M. mycoides* cluster, which can detect and identify Mmc and Mccp, but it requires two real-time PCR reactions.

HRM has been widely used for mutation scanning, methylation detection, mononucleotide polymorphism analysis, genotyping, sequence matching, and other applications because of its high sensitivity, good specificity, low cost, high-throughput detection. Douarre et al.^31^ described in 2012 a HRM-PCR assay for clearly differentiating the two main types of *Mycobacterium avium* subsp. *paratuberculosis* (cattle and sheep) and Gurtler et al.^32^ developed in the same year a HRM-PCR method for identification and van genotyping different *Enterococcus* species. In 2018, Liu et al.^33^ developed a highly specific multiplex high resolution melt-curve real-time PCR assay for the reliable detection of *Salmonella* serotypes. This approach is also suitable for establishing a method for rapidly identifying Mccp and Mmc.

In this study, the Mccp and Mmc sequences were compared to those published in NCBI to identify conserved gene fragments. A primer pair was designed using the Primer Premier 6.0 software to establish a HRM detection method for Mccp and Mmc. This method demonstrated specificity towards Mccp and Mmc without amplifying corresponding sequences from other common goat pathogens. Additionally, five positive samples detected using the HRM method were randomly selected for sequencing. The results showed that the sequence homology between the determined sequence and corresponding Mmc fragment was 100%, which demonstrated the high specificity of the method. The method showed strong reproducibility as melting curves of Mccp and Mmc were essentially the same as those of the positive control samples, and the intra- and inter-batch coefficients of variation for the two melting peaks were < 1%. Results showed that the minimum detection limits of Mccp- and Mmc-positive plasmids using this method were 55 copies/µL, and 58 copies/µL, respectively, whereas the minimum detection limit using SYBR GreenI qRT-PCR was 226 copies/µL and the detection limit with conventional PCR was 2.26 × 10^4^ copies/L^19^. Furthermore, the minimum detection limits of the multiplex PCR method were 4.03 × 10^5^ copies/µL and 6.78 × 10^5^ copies/µL for Mmc and Mccp, respectively^15^, indicating that the HRM method was more sensitive than the conventional and fluorescence qPCR methods for Mmc and Mccp detection.

To verify the practicability of this method in clinical testing of Mmc and Mccp, a total of 153 clinical samples were detected using the HRM method established in this study and compared to the results of fluorescence qPCR and *Mycoplasma* isolation and identification. The results show that the detection rate of Mmc using HRM was 20.3%, whereas that of SYBR GreenI qRT-PCR Mmc analysis and *Mycoplasma* isolation was 15.0% and 4.3%, respectively. Additionally, eight samples that were detected to be positive for Mmc with HRM method but negative by SYBR Green I qRT-PCR method were sequenced. The sequencing results confirmed the consistency with the corresponding fragment sequence of Mmc to be 100%, supporting the accuracy of Mmc detection using the HRM method, and indicating that the sensitivity of the HRM method was also higher than that of fluorescence qPCR and *Mycoplasma* isolation in detecting clinical samples. Furthermore, Thiaucourt^9^ established a real-time PCR method for detecting the *M. mycoides* cluster, which can detect and differentiate Mmc and Mccp, but it requires two separate fluorescence qPCR methods. The HRM method established in this study can differentiate Mmc and Mccp in only one reaction, which is faster and less labor-intensive. Because Mccp has not been found in Fujian Province, it was not detected in any of the clinical samples, which is consistent with the results of Jiang et al.^34^.

In summary, detection of Mmc and Mccp based on the HRM analysis established in this study can quickly and accurately distinguish the two pathogens with high specificity, sensitivity, and reproducibility. Compared to conventional and fluorescence qPCR methods, this method showed higher sensitivity and convenient operation. It can be used for rapid detection and identification of Mmc and Mccp in clinical nasal swab samples, rapid diagnosis, and epidemiological investigation of goat mycoplasmal pneumonia and contagious caprine pleuropneumonia.
